# Setting-related influences on physical inactivity of older adults in residential care settings: a review

**DOI:** 10.1186/s12877-017-0487-3

**Published:** 2017-04-28

**Authors:** Johanna G. Douma, Karin M. Volkers, Gwenda Engels, Marieke H. Sonneveld, Richard H.M. Goossens, Erik J.A. Scherder

**Affiliations:** 1 Clinical Neuropsychology, Vrije Universiteit Amsterdam, Van der Boechorststraat 1, 1081 BT Amsterdam, the Netherlands; 2 Industrial Design Engineering, TU Delft, Landbergstraat 15, 2628 CE Delft, the Netherlands; 3 Department of Neuroscience, Erasmus MC, Dr. Molewaterplein 50, 3015 GE Rotterdam, the Netherlands; 4 Center for Human Movement Sciences, University of Groningen, Antonius Deusinglaan 1, 9713 AV Groningen, the Netherlands; 5 Present address: Philadelphia Care Foundation, Amersfoort, the Netherlands

**Keywords:** Aged, Nursing home, Review, Sedentary lifestyle, Environment design, Caregiver

## Abstract

**Background:**

Despite the detrimental effects of physical inactivity for older adults, especially aged residents of residential care settings may spend much time in inactive behavior. This may be partly due to their poorer physical condition; however, there may also be other, setting-related factors that influence the amount of inactivity. The aim of this review was to review setting-related factors (including the social and physical environment) that may contribute to the amount of older adults’ physical inactivity in a wide range of residential care settings (e.g., nursing homes, assisted care facilities).

**Methods:**

Five databases were systematically searched for eligible studies, using the key words ‘inactivity’, ‘care facilities’, and ‘older adults’, including their synonyms and MeSH terms. Additional studies were selected from references used in articles included from the search. Based on specific eligibility criteria, a total of 12 studies were included. Quality of the included studies was assessed using the Mixed Methods Appraisal Tool (MMAT).

**Results:**

Based on studies using different methodologies (e.g., interviews and observations), and of different quality (assessed quality range: 25-100%), we report several aspects related to the physical environment and caregivers. Factors of the physical environment that may be related to physical inactivity included, among others, the environment’s compatibility with the abilities of a resident, the presence of equipment, the accessibility, security, comfort, and aesthetics of the environment/corridors, and possibly the presence of some specific areas. Caregiver-related factors included staffing levels, the available time, and the amount and type of care being provided.

**Conclusions:**

Inactivity levels in residential care settings may be reduced by improving several features of the physical environment and with the help of caregivers. Intervention studies could be performed in order to gain more insight into causal effects of improving setting-related factors on physical inactivity of aged residents.

**Electronic supplementary material:**

The online version of this article (doi:10.1186/s12877-017-0487-3) contains supplementary material, which is available to authorized users.

## Background

In 2012, *The Lancet* published a series of studies on physical activity, performed by the Lancet Physical Activity Series Working Group. One of these studies focused especially on physical inactivity and its negative influence on health, and even used the phrase ‘pandemic of physical inactivity’ [1]. This might be particularly true for older people, as physical activity levels may be lower for older adults than for younger people. In 2007, 21.6% of the U.S. noninstitutionalized older adults participated in regular leisure-time physical activity, compared to 37.1% of people aged 18-24 [2]. Regular leisure-time physical activity was defined here as a minimum of five times of half an hour of light or moderate intensity physical activity per week, or a minimum of three times 20 minutes of vigorous intensity physical activity per week. In addition to lower physical activity levels, more inactivity is observed in older adults. In 2007, 54.1% of the U.S. noninstitutionalized older adults were inactive, compared to 32.9% of people aged 18-24 [2]. Inactivity was defined here as not engaging in leisure-time physical activity of light or moderate, or vigorous intensity for a minimum of 10 minutes at a time, or an inability to engage in leisure-time activity. A recent review furthermore described that older adults spend 5.3-9.4 hours per day in inactive behavior [3].

Whereas physical activity has been shown to be associated with multiple health benefits [4], being physically inactive has several detrimental effects for older people. It can negatively influence cognition [5], contribute to a decline in physical function [6], and is considered a risk factor for several chronic diseases [7]. It even has a similar detrimental effect with regard to life expectancy as smoking and obesity [8].

Notwithstanding these detrimental effects of physical inactivity, within the aged population inactivity levels seem to be even higher in residents of a wide range of residential care settings (settings where older adults live other than the community) than in community-dwelling older adults. This was found, for example, for female residents of homes for the aged [9] and assisted care facilities [10], who were more sedentary than their community-dwelling counterparts. Women living independently were engaged in more active activities, such as household work, while institutionalized women spent more time in seated activities, such as reading [9]. When both genders were included, also less activity was recorded in residents of aged care facilities than in the older adults who lived independently [11]. In continuing care retirement communities or similar housing providers, lower levels of physical activity were found in nursing care than in assisted living, and lower levels of activity in assisted living than in independent living settings [12].

Even though some of the above-mentioned studies included only ambulatory residents, in general the higher level of physical inactivity in residential care settings than in the community might be due to a worse health condition of residents. Physical limitations have been mentioned most often as barrier to physical activity in at least one study [13]. Nursing home residents may suffer from diseases such as heart failure, a disease that often coincides with other diseases and with the use of multiple medications [14]. Polypharmacy is a known phenomenon among nursing home residents and is also related to, among others, ischemic heart disease, depression, and pain [15]. Pain is found to be related to physical inactivity as well [16].

Besides the difficult-to-modify factors described above, there may also be other, setting-related factors that could influence inactivity levels in residents of residential care settings. These factors include all aspects of a resident’s direct environment, such as the physical and social environment in the care setting. Such setting-related factors might be easier modifiable; therefore, if such factors become known, interventions can be directed towards decreasing detrimentally high levels of physical inactivity by making changes in the environment, possibly even apart from increasing residents’ participation in organized physical activity programs. An overview of the literature would be particularly useful to identify what setting-related factors might be addressed in order to reduce inactivity. However, to our knowledge, no previous review has focused specifically on setting-related reasons for ‘spontaneous’ inactivity (i.e., apart from participation in organized activities) of aged residents of residential care settings in all literature up to now.

### Aims

The aim of the present review is to review all studies that describe setting-related, environmental factors that are related to the level of physical inactivity of older adults living in a residential care setting.

## Methods

### Literature search

For this review, the PubMed, PsychINFO, Embase, Cinahl, and Cochrane databases were systematically searched for studies on physical inactivity of older adults in various care settings. The key words ‘inactivity’, ‘care facilities’, and ‘older adults’ were used, including their synonyms and MeSH terms. The term ‘activity’ or its synonyms were not included in the search, to have the focus specifically on *in*activity. The search terms for the five databases are shown in (Additional file 1). The search was conducted on 2 April 2012, and initially resulted in 5648 references. A search update using the same key words was conducted on 16 July 2015, and initially resulted in 2347 references.

### Study selection

After duplicates were removed, two reviewers (JGD and GE) independently evaluated the remaining titles and abstracts, based on the inclusion and exclusion criteria. Inclusion criteria were: ‘physical inactivity induced by the nursing home/residential care setting’ should be a main topic; papers needed to be written in English; should contain data on inactivity of aged residents (if no version of the word ‘elderly’ was used, an age of 65+ was considered old); have at least one ambulatory participant included; and describe ‘unneeded’ inactivity in humans. In order to obtain information on such unneeded inactivity, studies including *only* participants who were immobile (i.e., unable to be physically active), or with one or more specific disease(s) such as dementia (that might explain the inactivity to a certain extent) were excluded. Other exclusion criteria were: studies describing inactivity due to restraint use; and studies that were case studies, reviews, or intervention studies (except interventions in which changes in the environment were made, if these also contained useful baseline data). Discrepancies between evaluations were discussed, so that a joint decision could be made.

For all the remaining references, attempts were made to collect the full papers on physical inactivity in nursing homes and other residential care settings. The full papers were evaluated (JGD) to decide whether they met the above-mentioned eligibility criteria, and additionally, whether they studied and described setting-related factors that possibly influence physical (in)activity. If a paper met these criteria, it was included in the review. Additional full papers were also collected; these articles were selected from references used in articles included from the search, and were included if they met the criteria described above.

In order to include as many studies as possible on this subject, there were no restrictions concerning the study design, methods used to collect data on physical inactivity or the reasons for it, or to the applied definition of (in)activity, other than those described in the eligibility criteria. However, the (in)activity described had to be ‘spontaneous’, that is, not part of an organized (group) activity. Studies describing any other activity or lack of it could be included.

### Data collection and data analysis

The included papers were carefully searched for information on possible setting-related factors influencing physical inactivity. These factors were grouped into two main categories: 1) the influence of the physical environment; and 2) the influence of caregivers on the physical inactivity levels of residents. Study characteristics of all included studies were summarized under the heading ’Study characteristics’ of the Results section. This section also includes the results of the quality assessment (see below). The two subsequent sections describe the main results and conclude with a summary, in which the number of studies supporting the results is shown, as well as their assessed quality. Since articles were included irrespective of the applied definition of physical (in)activity, in this review the terms ‘inactivity’ and ‘activity’, as well as their synonyms, were used in their broadest sense, that is, not necessarily determined by set values such as guidelines or specific definitions for physical (in)activity.

### Quality assessment

Quality of the included studies was assessed using the Mixed Methods Appraisal Tool (MMAT) [17]. This tool is especially designed for assessing the quality of quantitative, qualitative, and mixed methods studies that are included in the same review. First, two screening questions need to be answered in order to assess the feasibility of using the tool for the study concerned. In addition, the study type is determined (which we based on the collected data for the study’s results section). Second, the quality of the study is assessed, using four questions for a quantitative study (different questions for randomized controlled, randomized non-controlled, and descriptive studies), or a qualitative study. For a mixed methods study, both the questions for the quantitative sub-domain and the qualitative domain need to be answered, as well as three questions on the mixed methods design. The final score for mixed methods studies is based on the score of the lowest-scoring section. For all types of studies, one asterisk (*) is indicative of a score of 25%, ** = 50%, *** = 75%, and **** = 100%. Quality assessment was performed by two researchers (JGD and RECSM), and discrepancies between scores were discussed. If no consensus was reached, a third researcher (GE) was asked for a third opinion on the item(s) concerned, in order to achieve a final quality score for all included studies.

## Results

### Study selection

After removing duplicates, and excluding references based on their title, abstract, setting, study type, and/or language, attempts were made to collect 51 full papers in the original search. This was possible for all but one paper, of which a full text could not be obtained; this study was therefore excluded. Based on the full texts, only seven of the remaining 50 papers met the criteria and were included. Two additional articles that were referred to in at least one of the seven papers were included as well, resulting in a total of nine papers that were included based on the original search.

**Fig. 1:**
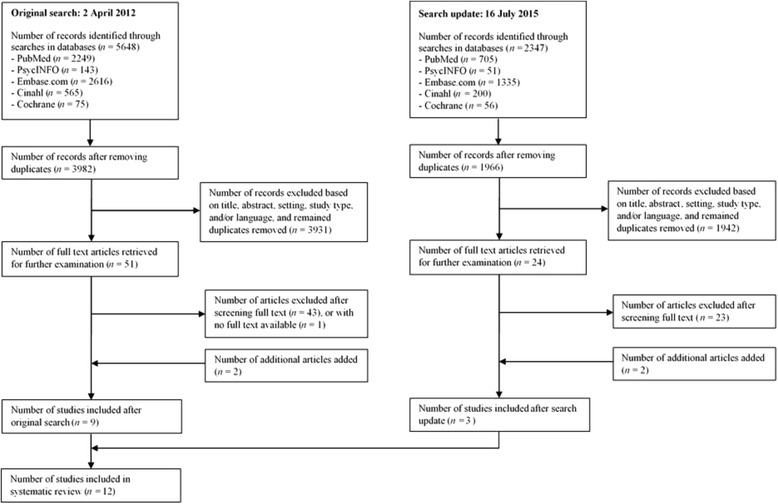
Flow diagram of study selection

For the search update, 24 full papers were collected, of which only one paper met all the eligibility criteria. Two additional papers were included that were referred to in this one paper, resulting in three papers that were added based on the search update. In all, 12 papers were included in this review. The selection process is shown in Fig. 1.

### Study characteristics

The study characteristics including the assessed quality of the 12 final papers are shown in (Additional file 2: Table S1). In total, five studies included information on influences of the physical environment, five studies on influences of caregivers, one study provided information on both factors, and one study provided information that could not be traced back to a specific factor. Based on the quality assessment, two studies (both qualitative) scored 100% and thus were assessed as meeting all criteria, four studies (two quantitative, two qualitative) scored 75%, four studies (three quantitative, one qualitative) scored 50%, and two studies (one mixed methods, one qualitative) scored 25%.

### Factors in the physical environment

With regard to the physical environment, first, a better person-environment fit (i.e., the fit between someone’s abilities and the demands of the environment, such as the right height of kitchen shelves for residents) is found to be related to more activity based on actigraphy activity counts [18]. This was not the case for the number of steps per day. These results were observed by examining residents of two different types of settings together. When the two types of settings were compared, both the functional ability and physical activity of residents were comparable, based on the ratings of caregivers. However, residents of the setting with, among others, a worse person-environment fit as regards the indoor environment were less physically active than residents in the setting with a better fit, based on actigraphy data.

Second, specific characteristics of the environment in terms of the layout of the building and it’s interior may influence inactivity levels. More specifically, a larger scaled building with more office space and other distances between several types of areas, improved accessibility features (e.g., greater corridor width), additional physical amenities, and the presence of a little more features concerning security (e.g., call buttons in the rooms of residents) may lead to a decrease in observed levels of passivity; a change that was observed when all residents of a nursing home moved to a new building [19]. In this study, interpersonal behavior increased. Another finding was that orienting became harder for the aged residents, due to the increased size and complexity of the setting. Even though there are more changes associated with a relocation than just the physical environment (e.g., in this study a third of the residents in the new building were new residents), the change in physical environmental features as measured by the Physical and Architectural Features Checklist from the Multiphasic Environmental Assessment Procedure was suggested as an important source of the change in behavior.

The security and accessibility and comfort of the physical environment may also specifically influence corridor walking [20]. Based on focus group discussions with residents of assisted living facilities, important aspects related to corridor walking regarding the security were good handrails, and carpet as the preferable type of floor covering. Concerning the accessibility and comfort, wide corridors, the possibility to sit, either long or short walking distances (depending on the reason for walking, i.e., for getting to destinations like the dining room or activity rooms, for exercising, or for social interaction), large elevator dimensions, and the accessibility of activity areas and restrooms were important. Negative aspects of walking indoors as compared to outdoors reported by residents were that there was a limited walking area (in some facilities), and that there were not as many things to see indoors as outdoors. Residents would enjoy corridor walking more if there were more aesthetically pleasing additions, such as artwork, plants, and windows [20].

Third, both the furniture and equipment present in residential care settings could also play a role in the physical inactivity of residents. In the rooms of less mobile older adults, beds were described in interviews with older adults as covering a large part of the room [21]. In addition, physical aids were often unfitting. Even though it was not specifically mentioned whether these aspects described for old people’s homes induced physical inactivity, this may actually be the case, as another interview study with older adults found that the readiness to be physically active decreases when a long-term care environment lacks equipment and appropriate areas for activities [13].

Fourth, based on surveys with managers and staff, one indoor facility (i.e., a physical therapy room), and two outdoor environmental features (i.e., a garden and a golf course) were found to be positively related to the percentage of residents in residential care settings who engaged in regular physical activity [12]. However, the number of indoor activity facilities did not seem to be significantly related to the percentage of residents who walked. The relationships between the presence of facilities/features in the physical environment and participation in several types of activities (possibly others than described here) were often found to be made weaker or even statistically non-significant by adding at least the covariate ‘number of available activity programs’. It was therefore proposed by the authors that an interplay between the physical environment, the organization, and characteristics of the resident could be more informative with regard to physical activity [12].

In sum, there are several physical environmental factors that seem to play a role in residents’ (in)activity levels. Most evidence is present for the influence of a range of specific characteristics of the environment, mainly those regarding safety and accessibility [19***, 20****]. A lack of suitable activity equipment and areas might also contribute to unneeded inactivity [13***, 21**]. Not only the actual environmental features seem to be important, but also their fit with the residents’ abilities [18**]. Finally, a few specific indoor or outdoor facilities may (possibly together with other factors) be related to activity levels of residents [12*].

### Caregiver-related factors

With regard to caregiver-related factors, first, the number of caregivers seems to play an important role in altering the level of physical inactivity. In one study, observations showed more inactivity by nursing home residents (of which some were rehabilitation patients) during weekends compared to weekdays, which co-occurred with fewer observed staff members for a greater number of observed residents in the weekends compared to weekdays [22]. Also with regard to the estimated amount of time residents of nursing homes spent in bed, more inactivity was observed in homes with lower staffing levels than in homes with high staffing levels [23]. In the latter study, division in the high- or low-staffing-home category was based on state cost reports and interviews with staff. Participants residing in homes with high staffing levels spent less time in bed (three hours during daytime) than participants residing in homes with lower staffing levels, who spent as much as five hours in bed during the day, both amounts of time being estimated averages. A facility’s staffing level was found to be the strongest predictor of observed in-bed times of residents [23].

Second, also the time caregivers have available may contribute to the amount of (in)activity of residents, a conclusion based on interviews with relatives of residents of residential care facilities. Caregivers have been perceived not to have sufficient time for individual residents [24, 25], which might be due to caring for more severely ill residents, but also to business with other tasks [24]. A lack of time can be a reason caregivers cannot go for a walk with residents [24, 25]. In addition, when caregivers have to do other tasks (e.g., cleaning) and are absent from the shared living/dining room [24], residents may sit there inactively or fall asleep [24, 25]. However, caregivers’ business may also lead to a delay in the time residents can be brought to bed [24].

Third, the amount and type of care given may be important. For example, based on interviews with aged residents of old people’s homes, caregivers may use ‘activating’ care by helping residents transferring from their beds to wheelchairs, but they may also bring residents to bed for a nap after lunch [21]. Caregivers may furthermore assist residents with walking: nursing homes seem to differ in the amount of help with walking residents receive from caregivers. In nursing homes with a higher proportion of bedfast residents based on scores on the Minimum Data Set bedfast prevalence quality indicator, participants were observed more time in bed; however, interviews with participants who were able to respond sufficiently to an interview also indicated that caregivers in these nursing homes provided them with more help with walking than was indicated for nursing homes with a lower proportion of bedfast residents [26]. The effect of walking assistance on the level of physical inactivity was not further described. However, providing help to residents who need assistance to some degree may be of importance, as another study in which older adults had been interviewed stated that the process of becoming bedfast can be delayed by coping with limited mobility in a positive way as well as by supplying the right help [21].

Caregivers of residential care facilities seem to differ in the way they approach residents. Based on interviews with relatives, some caregivers seem to do only what they should do, whereas others have a more active approach towards residents, in which they, for example, invite residents to help wiping the table [24].

Lastly, having nothing to do might also be a reason for nursing home residents to only sit during the day [27]. It remains unclear, however, if this reason derived from a limited number of the residents during interviews was related to a lack of social interactions, to a lack of organized activities, and/or possibly even to another factor.

In sum, with regard to the caregiver factor, lower staffing levels seem to be an important factor in residents’ physical inactivity levels [22**, 23***]; this aspect is supplemented with the finding that also the time that caregivers have available can contribute to (in)activity [24*, 25***]. Varying approaches of caregivers towards residents might be needed to be taken into account as well [21**, 24*]. As nursing homes differ in the amount of walking assistance provided [26**], it is important to realize that among others providing the right help can delay the process of becoming bedfast [21**]. Lastly, having nothing to do might be related to aged residents’ inactivity [27****; indefinite factor].

## Discussion

The aim of this review was to describe setting-related, environmental factors that are related to the level of physical inactivity of older adults living in residential care settings. To this end, a systematic search of the literature was performed, resulting in 12 eligible papers of different study types. Although a previous review reported several barriers to physical activity and restorative care programs [28], the current review adds to this important field by focusing specifically on setting-related reasons for ‘spontaneous’ physical inactivity (i.e., apart from participation in organized activity programs) in all literature up to now. The results suggest that several aspects of the physical environment may play a role in the level of physical (in)activity. This factor was supported by six studies [12*, 13***, 18**, 19***, 20****, 21**]. Furthermore, caregivers seem to be important for the amount of (in)activity; a factor that was supported by six studies [21**, 22**, 23***, 24*, 25***, 26**]. One other study provided additional information; however, it was unclear to what factor [27****].

Finding the physical environment and caregivers as environmental factors influencing physical inactivity levels is in line with previous literature. For example, both in an adult [29] and an older adult population [30], possible factors influencing physical activity can be clustered into three main factors. One of these main factors is the cluster ‘environmental factors’, consisting of the social and physical environment [29, 30]; two environmental factors that are similar to the ones that were reported in the present review. The other two main factors are the clusters ‘personal characteristics’ and ‘program or regimen-based factors’ [29, 30]. In another study, comparable influencing factors were proposed as reasons for inactivity in nursing homes, although clustered somewhat differently, namely patient-related, organizational (i.e., organized meaningful activities, and caregiver-related aspects), and environmental aspects [31]. In the present review, we purposefully focused on the environmental factors, including the physical and social environment, as these factors offer indications on how the regular daily environment of a residential care setting may contribute to the level of inactivity, and, consequently, how the level of inactivity may be changed through changes in this environment.

Outside the scope of the present review, there are indeed other, more individual factors that could influence residents’ level of physical (in)activity. One of these factors, obviously, is physical impairment or health problems [13]. In addition, some residents may be dependent on others to become activated if possible [24], which emphasizes the importance of the environment in lowering inactivity levels. A resident’s degree of satisfaction about living in a nursing home, depressive symptoms [32], risk of falling [33] and fear of falling, a history of inactivity, and too little knowledge of the beneficial effects of physical activity [13] are other examples of individual factors related to physical (in)activity. In addition, residents’ response to the environmental factors may also differ between residents. For example, besides the important role of caregivers in altering the amount of physical inactivity, residents’ attitude towards caregivers can sometimes play a role in the level of inactivity. Some people who need help to get out of bed, stay in bed some days, even though they think it is important to get up briefly, because they do not want to waste caregivers’ time and cause difficulty [21]. Residents may also differ in how they experience and value the nursing home setting. Some residents may want to have more to do in a nursing home, whereas others often value doing nothing and rest [27], possibly to look back on life [24, 27] and/or prepare for death [24]. Such reasons should naturally be taken into account.

Finally, also organizational aspects or a focus on activity in the care setting may play a role in (in)activity. For example, the relationship between the environment and activity participation may be influenced by the number of available organized activity programs [12]. In addition, for example, the found relationship between a physical therapy room in the environment and a higher number of residents that walk to their meals might co-occur with an emphasis on activity. A combination of several factors, including organizational aspects, may contribute to physical activity in residential care settings [12]. Most activities in a nursing home may be organized for the more active residents [34]. We argue that the focus should be particularly on those residents who are more inactive and less able to initiate activities by themselves, and offer them activities that they enjoy participating in.

### Study strengths and limitations

A few aspects should be taken into account in the interpretation of the results. In the present review, only inactivity and its synonyms were used as key words for the search, not activity and related synonyms. Even though this choice was made deliberately since the focus of this review was especially on setting-related influences on *in*activity in residential care settings because of its detrimental effects, it is possible that some studies were missed due to this decision. This may be especially the case for studies that focus on factors that influence *activity* levels; however, some of the included studies also provided insight into factors that are related to residents’ activity levels. Another aspect to consider relates to this review’s focus on ‘spontaneous’ physical (in)activity, as compared to activity due to participation in organized activities. However, the environment may influence presumably more planned/organized activities as well (e.g., swimming and aerobics) [12]. Furthermore, it should be noted that a decrease in inactivity does not always necessarily imply an increase in locomotion, but may also lead to more interpersonal behavior (also including receiving assistance from staff and participating in organized activity [19]).

A strength of this review is that it excluded studies that examined only participants with specific diseases such as dementia, or an inability to be physically active. By doing so, the focus could be on environmental factors that are related to (in)activity, rather than possible disease- or inability-related reasons for inactivity. However, some of the included studies even excluded people with cognitive impairment [13], or those with a certain level of cognitive impairment [26] for at least one measure. One could therefore question if the described influencing factors might apply to inactivity levels of nursing home residents in general, as by the time of nursing home admission, nearly 50% of older people already have dementia [35], and residents with dementia might have different levels of inactivity than residents without dementia (see [5]). However, many of the included studies did not seem to exclude residents with dementia or other specific diseases, and one study [24] even described only residents with cognitive and physical disabilities (not all with dementia) (E. Ericson-Lidman, personal communication, January 15, 2016). Furthermore, as physical limitations were no exclusion criterion, and, in addition, one of the included studies reckoned not having medical acuity data as a limitation [23], it should even be taken into consideration that physical conditions may still have played some role in the role of the environment in inactivity levels. Indeed, as older adults in residential care settings (especially men [36]) may have worsened physical function compared to their community-dwelling counterparts [37], the setting-related factors reported in this review may be considered additional, not completely isolated factors related to physical inactivity. This may however strengthen the generalizability of the findings. All this considered, with regard to the included residents, the present review is expected to provide a representative overview of environmental factors related to inactivity of aged residents of residential care settings.

This review is limited by the fact that only 12 studies of influences on physical inactivity were included. Most of these studies had a qualitative design (*n* = 6), followed by a quantitative non-randomized design (*n* = 3), a quantitative descriptive design (*n* = 2), and a mixed methods design (qualitative and quantitative descriptive; *n* = 1). As the studies vary in their ways of collecting data, also the source of data included in the results section of this review differed. From some studies, observation data was used [22, 23], from others information reported by older adults [13, 20, 21, 27], or by other persons about older adults [12, 23, 25], and from still other studies data derived from a combination of several sources was used [18, 19, 26]. On the one hand, this difference in data collection may strengthen the evidence, because similar findings were observed at least to some extent over these diverse studies. This seems to be true also for findings in various housing providers: similar findings with regard to specific environmental features were done in nursing home and assisted living facility settings, and, even though the influence of staffing level was found specifically in two nursing home studies, the importance of caregivers’ time was reported in residential care facilities and special housing facilities. It may hereby be of interest that the factor ‘influence of staffing levels’ was based on more objective, quantitative, data, whereas the factor ‘influence of caregivers’ time’ arose from interview data. On the other hand, the small number of studies that were mainly conducted in nursing home settings (as compared to e.g., assisted living facilities), the mainly qualitative methods, the limitation that only two studies scored 100% based on the quality assessment, the fact that most (*n* = 11) studies were from the U.S. or European countries, and also the finite controlling for possible confounders, make it important to interpret the results of this review study with caution. However, the results could incite experimental studies in which one of the described environmental factors is adjusted, while controlling for possible confounders, and examine the effect on the level of inactivity of aged residents.

Another limitation of this review could be that some of the included studies are rather old. One might argue that residents of care institutions are more active now, because there is more focus on activity programs in nursing homes [34], and that, therefore, reasons for inactivity in earlier years are now less relevant. However, passivity is not only reported in older studies [19, 38]; more recent studies also show high levels of inactivity [11, 18, 31]. As suggested elsewhere, it seems that the daily life of nursing home residents is still quite similar to the way it was decades ago [34]. We therefore argue that possible factors related to physical inactivity levels in earlier years can still be important nowadays.

### Recommendations

Based on the findings of the present review, encouraging aged residents of residential care settings to reduce inactivity by means of the environment could start with adjusting the physical environment in such a way that it better fits the competencies of the residents, improving accessibility and comfort (e.g., wider corridors with places to sit and larger elevator dimensions), enhancing safety (e.g., adding call buttons in the rooms of residents, good handrails in the corridors, and carpet as floor covering), improving the aesthetics (e.g., adding plants and artwork), adding relevant equipment, and possibly adding features such as a garden. In addition, residential care settings should have a sufficiently high staffing level. As caregivers’ available time seems to be of influence to decrease residents’ inactivity, if a higher number of staff is not possible financially, volunteers, visiting family, or other employees could be encouraged to, for example, go for a walk with the residents. The amount of help, but also a positive and activating attitude may be important to increase activity levels. Caregivers can motivate residents who are too inactive to decrease their inactivity levels, for example, by asking them to help with small household chores. As these recommendations are based on mainly qualitative studies of varying assessed quality, these hypotheses need to be examined in further intervention studies in order to determine if the changes indeed decrease physical inactivity in residential care settings.

It would be of interest to examine if the extent to which above-mentioned factors play a role differs for residents with different functional abilities, as this characteristic varied between participants in the included studies in this review. For example, the amount of assistance caregivers provide might be more important for more dependent residents. Increased understanding with regard to such aspects may be of even more importance because of the current trend for older adults to live independently longer, likely leading to lower functional ability levels in residential care settings. Furthermore, as caregiver-related factors accounted for about half of the information available on setting-related influences, it would be worthwhile to examine caregivers’ perspectives on residents’ physical inactivity. For example, caregivers’ fear of falling of older residents with dementia appears to be related to restraint use or activity restrictions [39]. The authors of that study suggest that it is important to take such fear into account when trying to optimize the use of these restrictions. However, in general little research seems to be conducted in the area of caregivers’ perspectives related to inactivity. Both the views of caregivers and the earlier-mentioned personal and organizational factors should preferably be taken into account when aiming to improve activity levels.

## Conclusions

Inactivity levels in residential care settings may be reduced by means of improving several features of the physical environment and with the help of caregivers. Intervention studies could be performed in order to gain more insight into causal effects of improving setting-related factors on physical inactivity of aged residents.

## Additional files

Additional file 1: “Search terms for review ‘Setting-related influences on physical inactivity of older adults in residential care settings: a review’”. It includes the search terms for the PubMed, PsychINFO, Embase, Cinahl, and Cochrane databases. (DOCX 20 kb)

Additional file 2: Table S1. "Study Characteristics of Included Studies on Residential-Care-Setting-Related Factors Related to Physical Inactivity of Aged Residents”. It includes the study characteristics of the included studies, including their assessed quality. (XLSX 16 kb)
