# Supplementary material for: Setting-related influences on physical inactivity of older adults in residential care settings: a review
Source: BMC Geriatr. 2017 Apr 28;17:97. doi: 10.1186/s12877-017-0487-3 (PMC5408383; doi:10.1186/s12877-017-0487-3)
Supplement: Supplementary file 1 — “Search terms for review ‘Setting-related influences on physical inactivity of older adults in residential care settings: a review’”. It includes the search terms for the PubMed, PsychINFO, Embase, Cinahl, and Cochrane databases. (DOCX 20 kb) [file 12877_2017_487_MOESM1_ESM.docx]

**Search terms for review ‘Setting-related influences on physical inactivity of older adults in residential care settings: a review ‘**

***PubMed***

1. inactivity[tiab] OR immobility[tiab] OR passive behaviour[tiab] OR passive behavior[tiab] OR passivity[tiab] OR "sedentary lifestyle"[MeSH Terms] OR sedentary[tiab] OR motionless[tiab] OR sitting[tiab] OR laying[tiab] OR "bed rest"[MeSH Terms] OR bed rest[tiab] OR passiveness[tiab] OR "immobilization"[MeSH Terms] OR immobilization[tiab]
2. "hospitals"[MeSH Terms] OR hospitals[tiab] OR hospital[tiab] OR academic medical centre[tiab] OR academic medical centers[tiab] OR academic medical center[tiab] OR academic medical centres[tiab] OR "hospital units"[MeSH Terms] OR hospital units[tiab] OR hospital unit[tiab] OR care facility[tiab] OR care facilities[tiab] OR residential facilities[tiab] OR residential facility[tiab] OR "nursing homes"[MeSH Terms] OR nursing homes[tiab] OR nursing home[tiab] OR skilled nursing facilities[tiab] OR skilled nursing facility[tiab] OR "assisted living facilities"[MeSH Terms] OR assisted living facility[tiab] OR assisted living facilities[tiab] OR "homes for the aged"[MeSH Terms] OR homes for the aged[tiab] OR home for the aged[tiab] OR care unit[tiab] OR care units[tiab] OR continuing care retirement community[tiab] OR continuing care retirement communities[tiab] OR care institution[tiab] OR care institutions[tiab] OR "hospital departments"[MeSH Terms] OR hospital department[tiab] OR hospital departments[tiab]
3. "aged"[MeSH Terms] OR aged[tiab] OR elderly[tiab] OR older adult[tiab] OR older adults[tiab] OR senior[tiab] OR seniors[tiab] OR old[tiab] OR older[tiab]
4. ((#1) AND #2) AND #3

***PsychINFO***

1. DE "Passiveness" OR ( TI "inactivity" OR TI "immobility" OR TI "passive behaviour" OR TI "passive behavior" OR TI "passivity" OR TI "sedentary" OR TI "motionless" OR TI "sitting" OR TI "laying" OR TI "bed rest" OR TI "passiveness" OR TI "immobilization" ) OR ( AB "inactivity" OR AB "immobility" OR AB "passive behaviour" OR AB "passive behavior" OR AB "passivity" OR AB "sedentary" OR AB "motionless" OR AB "sitting" OR AB "laying" OR AB "bed rest" OR AB "passiveness" OR AB "immobilization")
2. (DE "Nursing Homes" OR DE "Residential Care Institutions" OR DE "Hospitals" OR DE "Psychiatric Units" OR DE "Assisted Living" OR DE "Psychiatric Hospitals" OR DE "Intensive Care" OR DE "Psychiatric Units") OR (TI "hospitals" OR TI "hospital" OR TI "academic medical centre" OR TI "academic medical centers" OR TI "academic medical center" OR TI "academic medical centres" OR TI "hospital units" OR TI "hospital unit" OR TI "care facility" OR TI "care facilities" OR TI "residential facilities" OR TI "residential facility" OR TI "nursing homes" OR TI "nursing home" OR TI "skilled nursing facilities" OR TI "skilled nursing facility" OR TI "assisted living facility" OR TI "assisted living facilities" OR TI "homes for the aged" OR TI "home for the aged" OR TI "care unit" OR TI "care units" OR TI "continuing care retirement community" OR TI "continuing care retirement communities" OR TI "care institution" OR TI "care institutions" OR TI "hospital department" OR TI "hospital departments") OR (AB "hospitals" OR AB "hospital" OR AB "academic medical centre" OR AB "academic medical centers" OR AB "academic medical center" OR AB "academic medical centres" OR AB "hospital units" OR AB "hospital unit" OR AB "care facility" OR AB "care facilities" OR AB "residential facilities" OR AB "residential facility" OR AB "nursing homes" OR AB "nursing home" OR AB "skilled nursing facilities" OR AB "skilled nursing facility" OR AB "assisted living facility" OR AB "assisted living facilities" OR AB "homes for the aged" OR AB "home for the aged" OR AB "care unit" OR AB "care units" OR AB "continuing care retirement community" OR AB "continuing care retirement communities" OR AB "care institution" OR AB "care institutions" OR AB "hospital department" OR AB "hospital departments")
3. ( TI "aged" OR TI "elderly" OR TI "older adult" OR TI "older adults" OR TI "senior" OR TI "seniors" OR TI "old" OR TI "older" ) OR ( AB "aged" OR AB "elderly" OR AB "older adult" OR AB "older adults" OR AB "senior" OR AB "seniors" OR AB "old" OR AB "older" )

***Embase***

1. 'immobilization'/de OR 'sitting'/exp OR 'sedentary lifestyle'/exp OR 'bed rest'/exp OR 'inactivity':ab,ti OR 'immobility':ab,ti OR 'passive behaviour':ab,ti OR 'passive behavior':ab,ti OR 'passivity':ab,ti OR 'sedentary':ab,ti OR 'motionless':ab,ti OR 'sitting':ab,ti OR 'laying':ab,ti OR 'bed rest':ab,ti OR 'passiveness':ab,ti OR 'immobilization':ab,ti
2. 'hospital'/exp OR 'health care facility'/de OR 'nursing home'/exp OR 'assisted living facility'/exp OR 'home for the aged'/exp OR 'hospital department'/exp OR 'hospitals':ab,ti OR 'hospital':ab,ti OR 'academic medical centre':ab,ti OR 'academic medical centers':ab,ti OR 'academic medical center':ab,ti OR 'academic medical centres':ab,ti OR 'hospital units':ab,ti OR 'hospital unit':ab,ti OR 'care facility':ab,ti OR 'care facilities':ab,ti OR 'residential facilities':ab,ti OR 'residential facility':ab,ti OR 'nursing homes':ab,ti OR 'nursing home':ab,ti OR 'skilled nursing facilities':ab,ti OR 'skilled nursing facility':ab,ti OR 'assisted living facility':ab,ti OR 'assisted living facilities':ab,ti OR 'homes for the aged':ab,ti OR 'home for the aged':ab,ti OR 'care unit':ab,ti OR 'care units':ab,ti OR 'continuing care retirement community':ab,ti OR 'continuing care retirement communities':ab,ti OR 'care institution':ab,ti OR 'care institutions':ab,ti OR 'hospital department':ab,ti OR 'hospital departments':ab,ti
3. 'aged'/exp OR 'aged':ab,ti OR 'elderly':ab,ti OR 'older adult':ab,ti OR 'older adults':ab,ti OR 'senior':ab,ti OR 'seniors':ab,ti OR 'old':ab,ti OR 'older':ab,ti

***Cinahl***

1. ( (MH "Immobility") OR (MH "Life Style, Sedentary") OR (MH "Sitting") OR (MH "Bed Rest") OR (MH "Immobilization") ) OR TI ( "inactivity" OR "immobility" OR "passive behaviour" OR "passive behavior" OR "passivity" OR "sedentary" OR "motionless" OR "sitting" OR "laying" OR "bed rest" OR "passiveness" OR "immobilization" ) OR AB ( "inactivity" OR "immobility" OR "passive behaviour" OR "passive behavior" OR "passivity" OR "sedentary" OR "motionless" OR "sitting" OR "laying" OR "bed rest" OR "passiveness" OR "immobilization")
2. ( (MH "Academic Medical Centers") OR (MH "Hospital Units+") OR (MH "Hospitals") OR (MH "Cancer Care Facilities") OR (MH "Hospitals, Special") OR (MH "Hospitals, Psychiatric") OR (MH "Hospitals, Rural") OR (MH "Hospitals, Urban") OR (MH "Patients' Rooms+") OR (MH "Residential Facilities") OR (MH "Nursing Homes+") OR (MH "Health Facility Departments+") ) OR TI ("hospitals" OR "hospital" OR "academic medical centre" OR "academic medical centers" OR "academic medical center" OR "academic medical centres" OR "hospital units" OR "hospital unit" OR "care facility" OR "care facilities" OR "residential facilities" OR "residential facility" OR "nursing homes" OR "nursing home" OR "skilled nursing facilities" OR "skilled nursing facility" OR "assisted living facility" OR "assisted living facilities" OR "homes for the aged" OR "home for the aged" OR "care unit" OR "care units" OR "continuing care retirement community" OR "continuing care retirement communities" OR "care institution" OR "care institutions" OR "hospital department" OR "hospital departments") OR AB ("hospitals" OR "hospital" OR "academic medical centre" OR "academic medical centers" OR "academic medical center" OR "academic medical centres" OR "hospital units" OR "hospital unit" OR "care facility" OR "care facilities" OR "residential facilities" OR "residential facility" OR "nursing homes" OR "nursing home" OR "skilled nursing facilities" OR "skilled nursing facility" OR "assisted living facility" OR "assisted living facilities" OR "homes for the aged" OR "home for the aged" OR "care unit" OR "care units" OR "continuing care retirement community" OR "continuing care retirement communities" OR "care institution" OR "care institutions" OR "hospital department" OR "hospital departments")
3. (MH "Aged+") OR TI ( "aged" OR "elderly" OR "older adult" OR "older adults" OR "senior" OR "seniors" OR "old" OR "older" ) OR AB ( "aged" OR "elderly" OR "older adult" OR "older adults" OR "senior" OR "seniors" OR "old" OR "older" )

***Cochrane***

1. (“inactivity” OR “immobility” OR “passive behaviour” OR “passive behavior” OR “passivity” OR “sedentary” OR “motionless” OR “sitting” OR “laying” OR "bed rest” OR “passiveness” OR “immobilization”):ti,ab
2. ("hospitals" OR "hospital" OR "academic medical centre" OR "academic medical centers" OR "academic medical center" OR “academic medical centres” OR "hospital units" OR "hospital unit" OR “care facility” OR “care facilities” OR "residential facilities" OR "residential facility" OR "nursing homes" OR "nursing home" OR "skilled nursing facilities" OR "skilled nursing facility" OR “assisted living facility” OR “assisted living facilities” OR "homes for the aged" OR "home for the aged" OR “care unit” OR “care units” OR “continuing care retirement community” OR “continuing care retirement communities” OR “care institution” OR “care institutions” OR “hospital department” OR “hospital departments”):ti,ab
3. ("aged" OR "elderly" OR "older adult" OR “older adults” OR “senior” OR “seniors” OR "old" OR "older"):ti,ab
